# Cooled Multifunctional Platforms to Alleviate Heat Stress in Broiler Chickens: Effects on Performance, Carcass and Meat Quality Traits

**DOI:** 10.3390/ani14233448

**Published:** 2024-11-28

**Authors:** Bassem Khalfi, Kobe Buyse, Imad Khan, Camila Lopes Carvalho, Patricia Soster, Gunther Antonissen, Frank André Maurice Tuyttens

**Affiliations:** 1 Department of Veterinary and Biosciences, Faculty of Veterinary Medicine, Ghent University, 9820 Merelbeke, Belgium; kobe.buyse@ilvo.vlaanderen.be (K.B.); frank.tuyttens@ilvo.vlaanderen.be (F.A.M.T.); 2 Flanders Research Institute for Agriculture, Fisheries, and Food (ILVO), 9090 Melle, Belgium; 3 Department of Pathobiology, Pharmacology and Zoological Medicine, Faculty of Veterinary Medicine, Ghent University, 9820 Merelbeke, Belgium; imad.khan@ugent.be (I.K.); camila.lopescarvalho@ugent.be (C.L.C.); patricia.sosterdecarvalho@ugent.be (P.S.); gunther.antonissen@ugent.be (G.A.); 4 Poulpharm, Prins Albertlaan 112, 8870 Izegem, Belgium

**Keywords:** broiler chickens, environmental enrichment, cooled multifunctional platforms, heat stress, production performance, meat quality

## Abstract

Platforms have been shown to be a suitable environmental enrichment for broiler chickens, accommodating their motivation to roost and rest at an elevated position. In order to increase the animal welfare benefits, we designed prototype elevated platforms with additional functionalities: a local cooling system, a sheltered area underneath the platform and collection trays underneath the platform that prevent manure from falling on the litter. This study assessed the effects of these multifunctional platforms during thermoneutral and heat stress conditions on two key determinants of their commercial uptake potential, namely production performance, carcass and meat quality. In each of the three experimental rounds, 560 one-day-old male chicks (Ross 308) were equally assigned to four pens and reared for 43 days. The barn was divided into two compartments (thermoneutral and heat), each containing one enriched and one barren pen. To induce heat stress, the ambient temperature was increased to 32 °C during 6 h/day when the birds were 29–40 d old. The platforms did not compromise broiler performance. Heat stress negatively impacted body weight (*p* = 0.008), average daily gain (*p* = 0.009) and feed intake (*p* < 0.001) and improved the feed conversion ratio (*p* = 0.026). The platforms reduced mortality rate under heat stress but not under thermoneutral conditions (heat × enrichment *p* = 0.025), likely due to the cooling functionality. No major effects of the platforms on overall carcass and meat quality were observed, except for a reduction in the risk of breast muscle myopathies (*p* < 0.001), which could enhance consumer acceptance and improve profitability. To conclude, the use of cooled platforms under the conditions of this experiment mitigated the adverse effects of heat stress on mortality, reduced the risk of breast muscle myopathies and did not compromise any other production or carcass and meat quality parameters.

## Simple Summary

Considering the rising concerns about the impacts of heat stress and the need for higher welfare standards in broiler chicken production systems, this study explored the effects of enriching chicken houses with cooled multifunctional platforms on broiler performance and carcass and meat quality. These platforms were tested under both thermoneutral (22 °C) and heat stress (32 °C) conditions in an experiment with 560 chickens for each of the three repetitions. Heat stress adversely affected most performance parameters, except for the feed conversion ratio (FCR), which showed a slight improvement under heat stress conditions. The elevated platforms were associated with reduced mortality under heat stress and a reduced risk of breast muscle myopathies but did not seem to have affected any other production performance or meat quality parameter. These cooled platforms appear effective for mitigating the negative impacts of heat stress on the performance and meat quality of broilers.

## 1. Introduction

The environmental enrichment of broiler chicken houses with elevated structures has been shown to effectively promote broiler welfare. Such enrichment supports their motivation to express species-specific behaviours, such as perching, which is instinctively performed to avoid predators [1]. Different elevated enrichments, including straw bales [2], perches [3], and elevated platforms [4], have been investigated. However, broilers utilise these structures more effectively when the design is adapted to their physical capabilities. Broilers have difficulties maintaining their balance on traditional perches and show a preference for elevated platforms, which better accommodate their heavy body conformation. The access to these platforms is further improved when ramps are provided [5] and when ramps are not too steep. The presence of elevated platforms has been associated with significant benefits for broilers, including an increase in comfort behaviours, enhanced mobility, improved leg health, and a reduction in fear responses [6,7].

Given the importance of these elevated platforms, we developed prototypes of innovative multifunctional platforms with three functionalities added to basic elevated platform designs in order to enhance their positive impact on broiler productivity and welfare. These enhancements aim to address the following key husbandry challenges: (1) Heat stress as a major factor compromising broiler productivity, leading to reduced performance parameters such as body weight, body weight gain, and feed intake [8,9], as well as increased mortality rates [10,11]. It also adversely affects several meat quality parameters, including reduced carcass and meat yields [12,13]. Furthermore, heat stress induces physiological and behavioural changes, such as panting, wing spreading, increased restlessness, and reduced activity [14], ultimately leading to higher levels of discomfort and stress. To mitigate these effects, an integrated water-cooling system was incorporated into the platforms [15]. This system aims to alleviate heat stress through conductive heat transfer via footpad and skin contact. (2) The deteriorated litter quality and prolonged contact with moisture are often associated with welfare issues, including the development of footpad dermatitis, hock burns [16], and reduced plumage cleanliness [17]. To manage this problem, manure collection trays were installed beneath the platforms, preventing manure from falling on the litter and thereby improving litter conditions and associated welfare indicators. (3) The lack of shelters for resting and sleeping in intensive rearing systems can negatively impact the health, growth and welfare of broilers [18]. Additionally, the absence of protection structures often increases fear responses among the birds. To address this issue, a dark, sheltered space was created beneath the platforms to provide additional protection, mimicking the brooding behaviour of a mother hen and to reduce disturbances [19].

To facilitate the successful implementation of these prototypes in the poultry production sector, it is crucial to test the effects of these multifunctional platforms on broiler performance and meat quality. Therefore, the objective of this study was to investigate the effects of the multifunctional platforms on production performance, carcass characteristics and meat quality traits of broiler chickens under thermoneutral and heat stress conditions. We hypothesised that (1) enrichment with these platforms would not compromise the production performance, carcass characteristics or meat quality of broiler chickens, (2) heat stress would have a negative impact on most of the studied parameters and (3) that the platforms’ cooling system would mitigate these adverse effects.

## 2. Materials and Methods

### 2.1. Ethics Statement

All experimental procedures in this study were in compliance with the European guidelines for the care and use of animals in research (Directive 2010/63/EU) and were approved by the Ethical Committee of the Research Institute for Agriculture, Fisheries and Food (ILVO), Merelbeke, Belgium under authorisation number 2022/414. The research permit extended from 28 September 2022 until 31 December 2023.

### 2.2. Study Design

**Figure 1:**
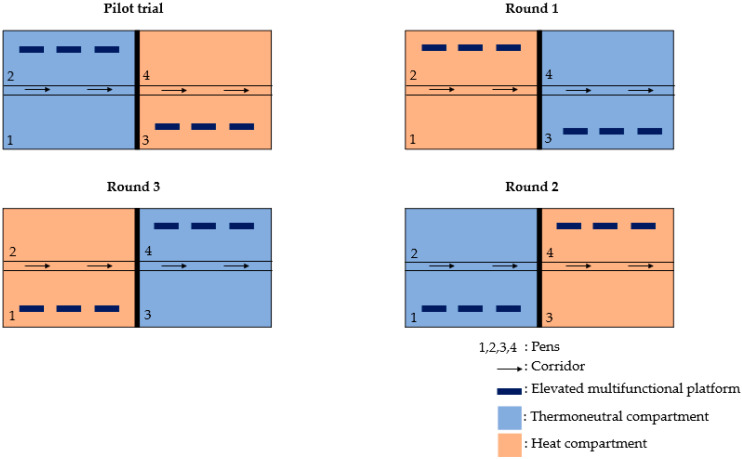
Experimental design and rotation plan between rounds.

**Figure 2:**
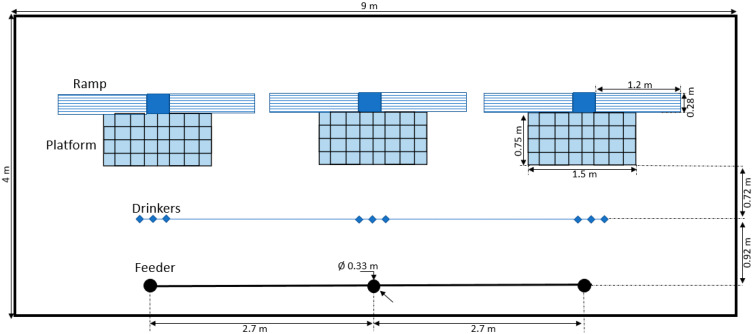
Top-view layout of an enriched experimental pen showing the dimensions and positions of the multifunctional platforms, drinker line and feeding line. The barren pens were identical, except that there were no platforms.

The experiment consisted of a pilot trial followed by the experiment proper, which included three consecutive rounds. All experiments were conducted in the same barn, which was divided into two compartments separated by a solid wall (Figure 1). In each compartment, there were two pens (9 × 4 m, Figure 2) either enriched with three multifunctional platforms (platform) or not (barren). After each round, the platforms were moved to the other pen within the same compartment. During each round and in alternating order, heat stress was induced in one of the compartments (heat) but not in the other compartment (thermoneutral). This resulted in a two-factorial block design with thermal conditions (heat stress vs. thermoneutral) as the first factor and enrichment (platforms vs. barren) as the second factor.

### 2.3. Animal Management

In each round, a total of 560 one-day-old male chicks (Ross 308) were purchased from a commercial hatchery (Belgabroed, Merksplas, Belgium) and reared for a period of 43 days at the ILVO research centre. The chicks were randomly assigned to the four pens (140 chicks/pen), resulting in a stocking density of 3.8 birds/m^2^. In this study, a lower stocking density was implemented, significantly lower than in standard commercial conditions (14–17 birds/m^2^), in order to comply with legislation to conduct experiments on animals and to accommodate other research objectives of the experiment. Wood shavings were used as bedding (2.5 kg/m^2^). The barn was a closed building, and temperature, air humidity, CO_2_, NH_3_ concentrations and lighting were regulated through the farm’s computer system in compliance with the standard rearing regulations (apart from the period when heat stress was induced). The farm’s computer system effectively regulated air ventilation, ensuring CO_2_ and NH_3_ concentrations remained within permissible levels. A dynamic LED lighting system (Explorentis, Leuven, Belgium) was used in this experiment as a part of another study to test light preferences. Three different light recipes were attributed for each functional zone in a balanced order similar for all pens as follows: Ref white 3000 k, Red and Cyan for the feeding line, Ref white 3000 k, Cyan and Yellow for the drinking line, Ref white 3000 k, Cold white 8000 k and Warm white 2000 k for the elevated platforms line and Ref white 3000k, White + UV and White + IR for the scratching line. The three light recipes were rotated between rounds. Since all pens were subjected to the same light recipes within a round, the outcomes of this study were not affected. During the first week, birds were maintained on a 23L:1D light program; from the second week until the end of the experiment, the light program was adjusted to 18L:6D. Birds were given food and water ad libitum and were fed with a standard commercial diet. A three-phase feeding regimen was followed with a starter (d1 to d11), grower (d12 to d24) and finisher diet (d25 to d43). The starter diet was a mash feed, whereas the grower and the finisher diets were pelleted feed. The nutritional composition of the diets varied across phases: crude protein levels were 204.64 g/kg in the starter, 195 g/kg in the grower, and 185 g/kg in the finisher diet. Metabolisable energy (ME) values were 2808.39 kcal/kg in the starter, 2854.83 kcal/kg in the grower, and 2951.80 kcal/kg in the finisher diet.

### 2.4. Elevated Multifunctional Platforms

**Figure 3:**
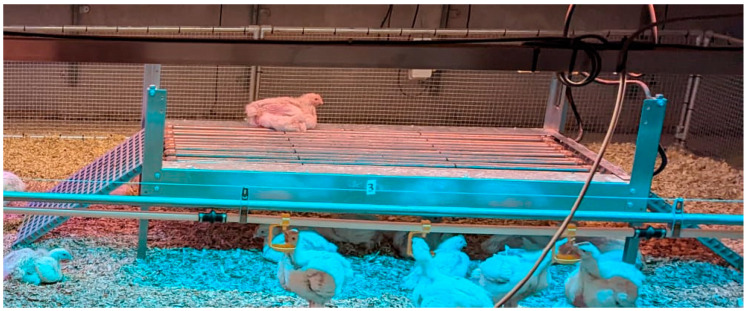
Preliminary platform design used during the pilot trial.

**Figure 4:**
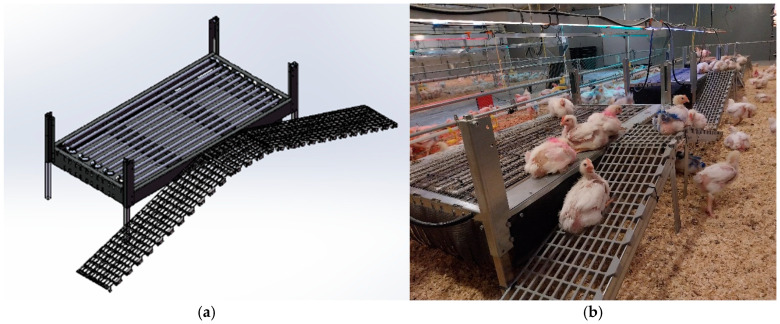
The updated elevated multifunctional platform: (**a**) three-dimensional (3D) design; (**b**) real picture.

The elevated multifunctional platforms (Roxell, Maldegem, Belgium) were made of steel and had a rectangular shape (1.5 × 0.75 m; Figure 3 and Figure 4). They were mounted at a height of 40 cm above the floor. The platform design used in the pilot trial (Figure 3) differed from the final design used in the experiment proper during rounds 1, 2 and 3 (Figure 4). During the pilot trial, we observed that the birds were not properly using the platforms, as the ramps were steep and not installed from d1, the resting area was uncovered (no mesh wire), and birds were perching directly on the cooling system tubes. These observations were taken into account to improve the design. Modifications were made to reduce the slope of the ramps from 25° to 18° using longer ramps, and a mesh wire was added to cover the upper part of the platforms, providing a more comfortable resting experience. Therefore, the results of the pilot trial were excluded, and the statistical analysis was based on the experiment proper with three rounds.

The updated platforms used in rounds 1, 2 and 3 (Figure 4) were covered on top by a wire mesh surface (mesh size 1.5 cm × 1.5 cm). Two plastic grid ramps (1.2 m × 0.28 m each; mesh size 19 mm × 40 mm) were attached to one side of the platforms, allowing the birds to move up and down the platform. The angle between the ramps and the barn floor was 18°. The upper part of the two ramps was connected to a horizontal transition area (274 mm × 246 mm) between the ramps and the platform. In the enriched pens, chicks had access to the elevated multifunctional platforms from d1 onwards. During the dark hours, the lights were maintained ON for an additional 15 min above the elevated platforms to encourage the birds to perch during darkness.

**Figure 5:**
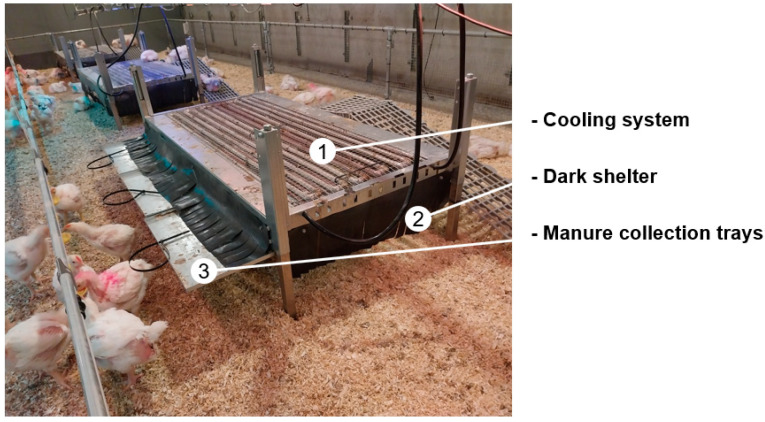
Additional functionalities of the elevated platforms.

In addition to the perching functionality, the elevated platforms were designed to provide three additional functionalities (Figure 5). (1) Cooling functionality with a local cooling system providing thermoregulation via the footpads and skin contact (e.g., when the belly and breast make contact with the platform as the bird sits down). Directly underneath and in direct contact with the wire mesh surface of the platform, there was a network of hollow galvanised metal pipes. The pipes system was connected to a water chiller (CNCTech, CW-5200 Industrial Chiller, China), and approximately 10 °C cool water was circulating in the system during heat stress. All elevated platforms in both compartments (thermoneutral and heat stress) were connected to this water-cooling system. (2) Dark shelter functionality to mimic somewhat the mother hen protection, the area underneath the platforms was accessible to the birds, and it was transformed into a dark, sheltered area (l × w × h: 0.75 m × 0.60 m × 0.40 m). The area was covered from all sides by black rubber (1 mm thickness), and some openings were made to create fringes that facilitate the entry and exit of birds. (3) Manure collection functionality involved collecting and weighing the manure of birds on the platforms using removable manure trays (3 per platform) installed below the grid surface. Manure was collected weekly or more frequently according to the accumulation of excreta.

### 2.5. Inducing Heat Stress

In the heat compartment, moderate chronic cyclic heat stress was induced between d29 and d40, applied twice over the course of five consecutive days.

The ambient temperature in the compartment was raised to 32 °C using the central heating system supplemented by an extra heater (Toolland, The Netherlands). Once the target temperature was reached, it was maintained for six hours before being gradually lowered to match the temperature in the thermoneutral compartment [20]. The warming-up period took two hours, and cooling down took three hours. During the heat stress, the temperature, the humidity and the CO_2_ levels were recorded hourly in the two compartments.

### 2.6. Data Collection

#### 2.6.1. Production Performance

The body weight (BW) (g) of all birds, as well as the given and leftover feed quantities (kg) at pen level, were measured at d1, d11, d24 and d40 using the scale (BAW-U4-1-500, Bascules Robbe, Belgium; accuracy: 1/150,000). At the start of the experiment and at the end of the starter, grower and finisher periods, respectively. Based on these data, the average daily gain (ADG) (g/bird/day), the average daily feed intake (ADFI) (g/bird/day) and the feed conversion ratio (FCR) were calculated for each growing phase and for the entire lifespan of the birds. Mortality was recorded daily for each group, and these records were used to calculate the mortality rate (%) and correct performance parameters for birds that died during the experiment.

#### 2.6.2. Carcass and Meat Characteristics

At the end of each production round, when the birds were 43d old, 32 birds per pen (384 in total) were randomly selected for the assessment of carcass and meat quality. The individual live weights (g) were recorded on the farm using the scale (DM-11000, UWE, Taiwan; accuracy: 1/11,000). Chickens were tagged and then transported to a commercial slaughterhouse. After slaughter, the birds were stored at 4 °C overnight. The following day, the eviscerated carcasses (without necks and giblets) were weighed, and carcass yields (%) were calculated using the formula:

(1) Carcass yield (%)=(Eviscerated carcass weight/ Live weight)×100

The carcasses were then cut to determine the weight of the following meat pieces: breast muscle (two fillets and two tenderloins), legs (two thighs and two drumsticks with skin and bones), wings and carcass remains (skin, fat tissues, bones). These weights were used to calculate the meat yield using the formula:

$$Meat\;yield\;\left(\%\right)=(Meat\;piece\;weight/\;Eviscerated\;carcass\;weight)\times 100$$

A pH-thermometer (HI98163 pH meter, electrode FC2323, Hannah Instruments, Temse, Belgium) was used to measure the meat pH and temperature, with 35 mm as aperture diameter and standard buffers (4 and 7) were used for calibration. A colourimeter (Miniscan EZ 4500L, HunterLab, Virginia, USA) was used to measure the meat colour (directional annular 45° illumination and 0° viewing angle). The colour measurement was based on the CIE (International Commission on Illumination) L* a* b* system. In this system, L* (lightness) represents the relationship between reflected and absorbed light, with a value of 100 for white and 0 for black. a* (redness) indicates redness when positive and greenness when negative, while b* (yellowness) reflects yellowness when positive and blueness when negative.

The pH, temperature and colour were measured at three different positions (top, middle and bottom) of the left breast muscles (384 samples in total). The same samples were used for the drip loss measurement using the EZ-DripLoss method (meat juice container method) [21]. The right breast muscles were evaluated for breast muscle myopathies, white striping and wooden breast (384 samples in total). A visual scoring scale was used to assess white striping, with a score of 0 (normal) indicating no distinct white lines, a score of 1 (mild) referred to small white lines, generally less than 1 mm thick but visibly apparent on the breast surface, a score of 2 (moderate) indicated larger white lines (1–2 mm thick), clearly visible on the surface and a score of 3 (severe) described thick white bands (>2 mm thick) covering most of the breast surface. Regarding the wooden breast, a scoring scale based on tactile assessment was used. A score of 0 (normal) when the breast was flexible throughout, a score of 1 (mild) when it was hard mainly in the cranial region but flexible elsewhere, a score of 2 (moderate) when it was hard throughout but flexible in the mid to caudal region, a score of 3 (severe) when the breast was extremely hard and rigid from the cranial region to the caudal tip [22,23].

Afterwards, the same right breast muscles were weighed, vacuum packed, and frozen (−20 °C) for four days. Then, the samples were thawed at room temperature for 16 h, after which they were removed from the vacuum bags, dried with a paper towel, and weighed to assess the thawing loss by subtracting the fresh weight (before freezing) from the thawed weight (after thawing). After measuring the thawed weights, the samples were vacuum packed again using their original bags, placed in a water bath of 80 °C and cooked for 45 min. Subsequently, the samples were taken out of the water bath, allowed to cool down at room temperature and dried with a paper towel, and the cooked weight (after cooking) was used to determine the cooking loss using two methods:

$$Cooking\;loss_{Thawed\;weight}\;(\%)=(Cooked\;weight/\;Thawed\;weight)\times 100$$

$$Cooking\;loss_{Fresh\;weight}\;(\%)=(Cooked\;weight/\;Fresh\;weight)\times 100$$

The breast muscles were then stored in a refrigerator (4 °C) for 24 h. Afterwards, 10 cylindrical samples (1.27 cm Ø) were taken from each fillet using a circular knife (parallel to the fibres’ direction). The 10 samples were placed beneath the cutting blade of a texture analyser (TA500 Lloyd Texture Analyzer fitted with a triangular Warner–Bratzler shear, Lloyd instruments, Bognor Regis, UK) to measure the mean shear force (N) for each fillet. The highest and the lowest shear force values per fillet were excluded, and the mean shear force was measured using the remaining 8 samples.

### 2.7. Statistical Analysis

The statistical analyses were performed using R studio (version 2023.6.2.0). The pen was considered the experimental unit for the entire trial, while the compartment was the experimental unit for the temperature factor during the finisher period. Data were analysed using least-square linear regression with enrichment (platform vs. barren) and temperature (heat vs. thermoneutral) as independent variables and the tested parameter as dependent variables. The interaction effect (when not significant) was removed from the final model to give more degrees of freedom to the main effects. Compartment and pen location were used as random effects. Cumulative logit models were used to assess ordinal breast myopathies scores, with the same fixed effects as described above. The number of observed myopathies with scores 2 and 3 was low; therefore, both scores were combined together for the analysis and classified as score 2. The data were checked for outliers and checked for a normal distribution on the residual. Differences were considered statistically significant at *p* < 0.05.

## 3. Results

### 3.1. Production Performance

There were no significant effects of the enrichment and temperature interaction on any of the performance variables (BW, ADG, ADFI, FCR), with the exception of the overall mortality rate (Table 1). The effect of heat stress on mortality depended on the presence of the platforms; under thermoneutral conditions, mortality was higher when platforms were present, but during heat stress, mortality was lower when platforms were present. Apart from a slightly higher FCR during the grower phase, the platforms had no effect on any of the performance variables. Heat stress, however, reduced BW, ADFI, ADG, and FCR, regardless of the presence or absence of platforms.

### 3.2. Carcass and Meat Quality Traits

**Figure 6:**
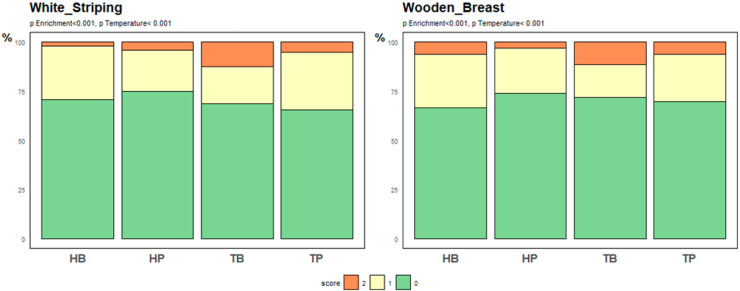
Effects of enrichment and temperature on broiler chickens breast muscle myopathies (TB: Thermoneutral-Barren, TP: Thermoneutral-Platform, HB: Heat-Barren, HP: Heat-Platform).

None of the carcass and meat quality parameters were affected significantly by the interaction between enrichment and temperature, with the exception of the pH measure (Table 2). Heat slightly increased the pH, but only when no platforms were present. The platforms had no effect on any of the other meat quality traits, apart from a non-significant trend for a small reduction in breast yield. Chickens exposed to heat stress showed higher drumstick yields and a non-significant tendency for increased carcass remains yields. Regarding the two breast muscle myopathies (white striping and wooden breast), lower scores were recorded under heat stress conditions and when platforms were present (all *p* < 0.001, Figure 6).

## 4. Discussion

The environmental enrichment through the elevated multifunctional platforms did not compromise the performance or meat quality of broiler chickens. Promising results were observed, as access to these platforms lowered mortality rates under heat stress conditions and reduced the risk of breast muscle myopathies.

### 4.1. Production Performance

This study showed that the provision of the elevated platforms had no effects on most of the performance variables, and higher FCR during the grower phase was recorded for the enriched group. The higher FCR could indicate that broilers expended more energy, possibly due to increased movement or activity levels associated with accessing the platforms. These findings contrast with the results of Jonas et al. [2], where elevated platforms were associated with increased weight gain and feed intake without any significant impact on FCR or mortality.

Under heat stress, we observed a pronounced drop in feed intake. This explains the lower body weight and average daily gain observed in our study in agreement with the findings of Goo et al. [9] and Wang et al. [24]. Heat-stressed birds reduce their feed intake as a mechanism to lower the increased metabolic heat production [13,25], and they release more stress hormones, which reshape the nutrient metabolism in the intestines [24]. Through this process, broilers lose productive energy, leading to an energy deficit that is not conducive to growth. In contrast with earlier studies where heat stress was linked to a higher FCR [26,27], in the present study, the heat stress resulted in a lowered FCR (finisher and overall). There is a possibility that the heat stress induced in our study was not sufficiently severe or prolonged for the reduction in feed intake to lead to a proportional reduction in growth.

An interesting interaction effect was documented for the overall mortality rate, which was reduced with the presence of platforms under heat stress but not under thermoneutral conditions. This positive interaction could be explained by the thermoregulation effect of the platform’s cooling system, resulting in a lower mortality rate under high ambient temperatures. Some caution is warranted, though, as the sample size of the present experiment was rather limited for reliable mortality estimates.

### 4.2. Carcass Characteristics and Meat Quality

The carcass and meat yields were not influenced by the presence of the elevated platforms, which is consistent with the results of Bench et al. [28] and Fidan et al. [3], where perches were provided as elevated enrichment. Meat pH is one of the most important meat quality traits since it directly affects other traits such as meat colour, water-holding capacity, cooking loss, juiciness and shelf life [29]. Under thermoneutral conditions, we documented a higher meat pH for the platforms group. The higher meat pH observed when platforms were provided could be explained by the improved overall environmental conditions, which likely promoted a more favourable meat pH. More comfortable, less stressed birds exhibit less glycogen depletion, leading to less synthesis of lactic acid, thereby maintaining a higher pH. Increased meat pH would reduce the meat water losses and reduce the risk of pale soft exudative meat condition [30]. Our results are in contrast with the findings of Karaarslan et al. [31], where perches did not affect meat pH.

Increasing broiler activity levels with perches has been shown to increase meat redness as a consequence of the higher myoglobin storage in the muscles [32]. However, our study showed no effects of elevated platforms on meat colour, cooking loss, or drip loss, aligning with the findings of Karaarslan et al. [31], Fidan et al. [3], and Kiyma et al. [33], respectively, and all these studies included perches as enrichment. Previous studies revealed that broilers with higher activity levels when given outdoor access, showed higher shear force and toughness of meat [34,35]. Nevertheless, no significant effects were recorded regarding the shear force with the provision of platforms in this study. The occurrence of breast muscle myopathies, such as white striping and wooden breast, poses a significant threat to the poultry meat industry by reducing consumer acceptance and leading to severe economic losses. These myopathies are a result of the high genetic selection of broiler chickens for faster growth and increased breast yields, which has also raised consumer concerns about animal welfare standards within the meat production industry [22,36,37]. According to Mutryn et al. [38], the increased activity could contribute to higher oxidative stress and hypoxia in the muscles, potentially raising the risk of myopathies. However, in this study, elevated platforms were found to have significantly reduced the incidence of breast muscle myopathies. It is possible, then, that the level of activity induced by the platforms was not a risk factor for myopathies development. This raises another research question, as the extent to which these platforms increased the birds’ activity levels remains uncertain and is still being investigated. This study suggests that the platforms are a promising approach to balancing animal welfare and meat production efficiency. As the demand for higher welfare standards grows, incorporating these platforms can give producers a competitive edge by reducing economic losses from meat myopathies and meeting consumer expectations.

Regarding the effects of heat stress, the results showed that the drumstick yield was positively affected by heat stress, in accordance with other studies reporting higher leg yield after heat stress exposure [10,39]. This result could be explained by reduced feather proportion in order to improve heat losses, as reported by Geraert et al. [40]. Slightly higher carcass remains yields, including abdominal fat, were also expressed under heat stress. This could be explained by the disruption of lipolysis, lipolytic enzyme activity and lipid metabolism-related pathways during heat stress exposure, which subsequently resulted in more abdominal fat deposition for the heat-stressed broilers, as reported by Lu et al. [39].

We expected that the exposure to heat stress would have reduced the meat water holding capacity, leading to higher moisture levels and increased drip and cooking losses, as reported in the studies of Zhang et al. [12] and Lu et al. [41]. However, no effects were observed for cooking loss and drip loss. It was anticipated that the exposure to heat stress would have reduced meat shear force, as expressed in the study of Lu et al. [41], due to the denaturation and disruption of the sarcomere structure [42]. Nevertheless, in the current study, heat stress did not affect the shear force. Heat-stressed birds exhibited lower body weights and fewer breast muscle myopathies, likely due to their reduced growth rate, which has been associated with a lower risk of myopathies, according to previous studies [37,43].

It is possible that the induced heat stress was not prolonged or severe enough to affect more meat quality parameters, such as drip loss, cooking losses and shear force, implying that the mitigating effect of the cooling platforms could also not be fully demonstrated. However, we opted for shorter but repeated periods of heat stress (32 °C, 6 h/day) instead of prolonged heat stress to minimise the risk of potential consequences of heat stress on animal health and welfare. In addition, the heat stress schedule of the current experiment was based on previous research at ILVO [20], which was found effective for inducing heat stress. This choice balanced animal welfare concerns with the objectives of the experiment and complied with ethical guidelines.

In the current study, heat stress was induced at the end of the rearing phase, considering that broilers are more susceptible to environmental stress during the final rearing stages, when issues related to high environmental temperatures often arise, such as relevant increases in body temperature and respiratory rate, important behavioural changes, reduced performance and increased mortality. Besides, by this age, broilers are sufficiently habituated to access the platforms, making it an optimal period to evaluate the effectiveness of cooling platforms and assess their impact on animal welfare and performance under heat-stress conditions.

The combination of the three additional functionalities (cooling system, manure collection, and dark shelter) makes it challenging to attribute the observed effects to a specific functionality. However, the current experiment was mostly designed to evaluate the effects of the platforms under thermoneutral vs. heat stress conditions to explore mainly the potential effect of cooling functionally and how it would alleviate the impacts of heat stress on performance, meat and carcass quality of broilers. Based on the promising results of the current experiment, we intend to test the functionalities of the platform separately in follow-up studies under commercial circumstances.

Additionally, testing these platforms under commercial conditions is crucial to improve the prototype design. While the current prototype platforms were effective for testing the concept in this experimental trial, further optimisation is required for their commercial uptake, such as incorporating automated manure belts and simplifying cleaning and removal processes after production rounds. Finally, caution is necessary when extrapolating these findings to commercial practices, as the conditions of this experiment differed substantially from commercial ones. For instance, in this study, a low stocking density was used, and only male birds were included, whereas commercial systems typically involve high stocking densities and combined sexes.

## 5. Conclusions

The present study revealed that environmental enrichment through the provision of cooled multifunctional platforms did not compromise the production performance or carcass and meat quality of broiler chickens. Heat stress adversely affected most of the performance variables except the feed conversion ratio. Access to these elevated platforms lowered mortality rates under heat-stress conditions and reduced the risk of breast muscle myopathies, likely due to the cooling functionality. Given these promising results, further research should focus on developing the platform prototypes to be more suitable for commercial uptake. Additional studies are needed to evaluate the effects of these platforms under commercial conditions, including the assessment and optimisation of each functionality separately with respect to animal welfare, performance, and environmental impact. For instance, the optimal temperature setting for water cooling, depending on the age of the birds and the ambient temperature, needs to be investigated.

## Figures and Tables

**Table 1:** Effects of enrichment, temperature and their interaction on broiler chickens’ production performance during the starter, the grower and the finisher periods.

	Thermoneutral		Heat		*p*-Value			SEM
	Barren	Platform	Barren	Platform	Enrichment (E)	Temperature (T)	Interaction (E × T)	
BW								
Day 1	43.39	43.49			0.812			0.58
Day 11	237.62	232.28			0.110			6.69
Day 24	951.46	939.04			0.189			20.43
Day 40	2350.01	2358.34	2294.12	2274.49	0.693	0.008	0.332	63.27
ADG								
Starter	19.42	18.87			0.106			0.62
Grower	54.91	54.36			0.331			1.18
Finisher	86.53	87.19	84.78	84.98	0.513	0.059	0.748	2.73
Overall	59.14	59.33	57.71	57.22	0.680	0.009	0.345	1.61
ADFI								
Starter	24.39	23.83			0.447			0.69
Grower	70.86	71.02			0.858			1.58
Finisher	133.22 ^a^	134.31 ^a^	127.88 ^b^	128.89 ^b^	0.232	<0.001	0.966	1.90
Overall	84.88 ^a^	85.66 ^a^	81.99 ^b^	81.89 ^b^	0.635	<0.001	0.557	1.43
FCR								
Starter	1.25	1.27			0.713			0.02
Grower	1.29 ^b^	1.30 ^a^			0.002			0.01
Finisher	1.55	1.55	1.51	1.52	0.821	0.009	0.827	0.03
Overall	1.43	1.44	1.42	1.43	0.136	0.026	0.994	0.02
MR								
Starter	0.11	0.73			0.115			0.26
Grower	0.84	0.72			0.756			0.19
Finisher	2.47	3.06	2.23	0.97	0.662	0.322	0.208	0.56
Overall	2.96	5.06	3.73	1.94	0.146	0.569	0.025	0.63

Means in the same row and with different letters are significantly different (*p* < 0.05). SEM: standard error of means; BW: body weight (g); ADG: Average daily gain (g/bird/day); ADFI: Average daily feed intake (g/bird/day); FCR: Feed conversion ratio; MR: Mortality rate (%); Starter: d1–10; Grower: d11–24; Finisher: d25–40; Overall: d1–40.

**Table 2:** Effects of enrichment, temperature and their interaction on broiler chickens carcass and meat quality characteristics.

	Thermoneutral		Heat		*p*-Value			SEM
	Barren	Platform	Barren	Platform	Enrichment (E)	Temperature (T)	Interaction (E × T)	
Carcass traits								
Carcass (%)	67.24	67.01	66.93	67.24	0.818	0.711	0.857	0.28
Breast (%)	29.48	29.31	29.08	28.51	0.073	0.226	0.327	0.12
Drumstick (%)	14.21	14.16	14.33	14.70	0.275	0.023	0.108	0.07
Thigh (%)	27.72	27.77	27.80	28.05	0.301	0.210	0.470	0.07
Wings (%)	11.10	11.16	11.15	11.19	0.620	0.708	0.919	0.05
CR (%)	11.62	11.82	11.85	11.92	0.153	0.059	0.353	0.42
Meat quality								
L*	57.98	57.36	57.48	57.74	0.604	0.868	0.175	0.11
a*	6.33	6.18	6.16	6.14	0.380	0.714	0.484	0.06
b*	14.79	14.69	14.51	14.61	0.99	0.554	0.630	0.06
TL (%)	6.17	6.92	5.45	6.50	0.495	0.894	0.259	0.22
CL/TW (%)	28.22	27.14	27.34	27.24	0.177	0.462	0.252	0.22
CL/FW (%)	32.63	31.47	31.39	32.63	0.658	0.576	0.170	0.25
Drip loss (%)	1.62	1.52	1.52	1.39	0.330	0.334	0.925	0.06
Shear force (N)	6.78	6.96	6.21	7.11	0.223	0.634	0.428	0.07
Temp. (°C)	5.1	4.95	5.28	4.89	0.106	0.793	0.548	0.09
pH	5.81 ^b^	5.86 ^a^	5.87 ^a^	5.83 ^a^	0.042	0.005	0.001	0.01

Means in the same row and with different letters are significantly different (*p* < 0.05). SEM: standard error of means. CR: Carcass remains (%); TL: Thawing loss (%); CL/TW: Cooking loss based on thawed weight (%); CL/FW: Cooking loss based on fresh weight (%); Temp.: Temperature (°C).

## Data Availability

All available data are incorporated in the manuscript.
